# Effects of prenatal oral l-arginine on birth outcomes: a meta-analysis

**DOI:** 10.1038/s41598-021-02182-6

**Published:** 2021-11-23

**Authors:** Eita Goto

**Affiliations:** Department of Medicine and Public Health, Nagoya Medical Science Research Institute, 1-118 Kamenoi, Meitou-ku, Nagoya, 465-0094 Japan

**Keywords:** Health care, Medical research, Signs and symptoms

## Abstract

Adverse birth outcomes are associated with elevated mortality and morbidity rates throughout life. This meta-analysis of randomised controlled trials examined whether prenatal oral l-arginine has effects on birth outcomes. A total of 45 overall good quality studies were extracted from 10 finally eligible articles. In comparison to controls, providing oral l-arginine to women with a history of poor pregnancy outcomes significantly reduced risks of intrauterine growth retardation neonates, pre-term birth and respiratory distress syndrome (*n* = 7, 3 and 3, respectively) and significantly increased birthweight and gestational age (*n* = 8 and 5, respectively) l-Arginine significantly increased Apgar score in women at high risk of pre-eclampsia or with pre-eclampsia or gestational or mild chronic hypertension in comparison to controls (*n* = 4). l-Arginine showed no significant effect on any other outcome examined (*n* = 2). The quality of evidence was at least medium or high. Consequently, oral l-arginine may be at least moderately recommended for women with a history of poor pregnancy outcomes and at high risk of pre-eclampsia or with pre-eclampsia or gestational or mild chronic hypertension. However, further studies are required to provide stronger conclusions, partly due to small study effects.

## Introduction

Adverse birth outcomes, such as intrauterine growth retardation (IUGR), low birthweight, pre-term birth and low Apgar score, are associated with elevated mortality and morbidity rates in the neonatal period as well as in adulthood^1^. Prenatal oral supplements to improve birth outcomes typically include iron folic acid, multiple micronutrients and lipid-based nutrients. Lipid-based nutrients have the greatest efficacy in improving birth outcomes^2^. On the other hand, placental insufficiency (or uteroplacental vascular insufficiency) may increase the risks of low birthweight, IUGR, pre-term birth and stillbirth^3–6^. The amino acid, l-arginine, which is nutritionally essential for the foetuses^7^, given to pregnant women has been proposed to reduce these adverse birth outcomes due to its role in the synthesis of nitric oxide, which acts as a mediator of vascular relaxation and inhibits platelet adhesion^8^. The results with inclusion of data for both intravenous and oral l-arginine given to women suggested that l-arginine increased gestational age and birthweight in women with IUGR foetuses^9^. Although oral administration is easier, less expensive and more convenient than intravenous l-arginine, the evidence for beneficial effects of oral l-arginine alone in pregnant women with and/or without risk factors for adverse birth outcomes is still inconclusive. It is likely that further studies have since been published regarding the effects of oral l-arginine alone on birth outcomes in women with varying background characteristics.

The present meta-analysis was performed to determine whether prenatal oral l-arginine has favourable effects on birth outcomes.

## Methods

All methods were carried out in accordance with relevant guidelines and regulations.

### Effect measures and eligibility criteria

The effect measures considered in this meta-analysis were relative risks of IUGR neonates, i.e., fetal weight or birthweight < 10th centile, low birthweight or underweight, i.e., birthweight < 2500 g, pre-term birth, stillbirth, abortion, perinatal or neonatal death, stunting, infection, respiratory distress syndrome (RDS), intracranial haemorrhage (ICH), neonatal intensive care unit (NICU) admission and Caesarean section (dichotomous outcomes) and mean differences in birthweight, birth, crown-to-rump, crown-to-coccyx, sternal, foot, femur or sole length, head, chest, arm, abdominal, thigh or calf circumference, biparietal diameter, subscapular, biceps or triceps skinfold thickness, body mass index, Ponderal index, weight-for-age z-score, length-for-age z-score, weight-for-length z-score, gestational age and Apgar scores at 1 min and 5 min (continuous outcomes) between pregnant women given oral l-arginine (l-arginine group) and those given other or no supplements (control group). The effect measures included those according to which peri- and pre-conceptional strategies to reduce maternal and neonatal mortality and morbidity rates may have differed and neonatal anthropometries that may have reflected their nutritional, metabolic or genetic status associated or possibly associated with their elevated mortality and morbidity rates. The inclusion criteria were English language randomised controlled trials (RCTs) of singleton pregnancies that provided: (a) the numbers of mother–neonate pairs with and without dichotomous outcomes in each group or (b) the number of mother–neonate pairs in each group and mean differences in continuous outcomes and their standard deviations between the two groups.

### Information sources, search strategy and study selection process

The electronic databases searched were PubMed (MEDLINE), ClinicalTrials.gov, CINAHL, PsycINFO, Wiley Online Library, ProQuest Central (e.g., ProQuest Health and Medical Complete and ProQuest Nursing & Allied Health Source), ProQuest Dissertations & Theses Global, the entire Cochrane Library (e.g., CENTRAL), Web of Knowledge, Google Scholar and Sage Publication Online (November, 2019). The terms described in the ‘PubMed search strategy’ subsection of the Supplementary Methods were used to search PubMed (MEDLINE) with no restrictions regarding publication date. The titles and abstracts of articles were scanned to identify unrelated articles that were then excluded. Those that remained were selected for inclusion in the analysis. The full texts of the selected articles were retrieved to identify additional unrelated articles that were then excluded. The remaining articles were considered as potentially eligible articles. PubMed Related Citations shown by clicking the tabs entitled ‘See all…’ (old version) at the right sides on the screens of the potentially eligible articles, bibliographic references of the potentially eligible articles and the articles displayed using other databases were also investigated. Reviews, Letters to the Editor, meeting précis and other articles reporting studies that did not provide the primary data were excluded. This process was repeated periodically. Duplicate publications were merged.

### Data collection process and data items

The characteristics of the included studies—i.e., first author names, publication dates, countries, populations, outcomes, contents of interventions, e.g., amounts of l-arginine, contents of controls, and the numbers of mother–neonate pairs with and without dichotomous outcomes among l-arginine and control groups or the numbers of mother–neonate pairs among l-arginine and control groups, mean differences in continuous outcomes between these two groups, and their standard deviations—were extracted. Studies were grouped according to the following categorizations of these characteristics: (a) study region, i.e., Africa, Asia, Europe, Latin America, the Middle East, North America or Oceania vs. others and developing vs. developed countries; (b) population, i.e., including vs. excluding (high risk of) pre-eclampsia or gestational or mild chronic hypertension and including vs. excluding (symmetric or vascular) IUGR foetuses; (c) amounts of l-arginine, i.e., 3 g per day vs. > 3 g per day; (d) contents of controls, i.e., vitamins or placebo vs. none; (e) time of Apgar score, i.e., 1 min vs. 5 min after delivery; and (f) study quality, i.e., ‘(probably) yes’ vs. ‘(probably) no’ responses to questions of a revised Cochrane risk-of-bias tool for RCTs (RoB 2)^10^ and low risk-of-bias vs. some concerns regarding risk-of-bias or high risk-of-bias (See ‘Study Risk of Bias Assessment’).

### Study risk of bias assessment

Study quality was assessed using a revised Cochrane risk-of-bias tool for RCTs (RoB 2)^10^. RoB 2 uses three questions regarding ‘random sequence generation’, ‘allocation concealment’ and ‘baseline difference’ and evaluated ‘risk-of-bias’. Quality assessment using RoB 2 was performed five times, and the most frequent responses were selected as the final responses.

### Synthesis methods and certainty assessment

The following statistical analyses were performed using Stata/MP 13.1 (StataCorp LP, College Station, TX, USA). Substantial heterogeneity was defined as *I*^2^ ≥ 50%^11^. Meta-analysis was performed to summarise relative risks of dichotomous outcomes and mean differences in continuous outcomes. A fixed-effects model (the method of inverse variance) and a random-effects model (the method of DerSimonian & Laird) were used to summarise the data with *I*^2^ = 0% and *I*^2^ > 0%, respectively^12,13^. The 95% confidence interval and 95% prediction interval (i.e., the region where studies in the future will fall within 95% probability) calculated using a random-effects model were shown in the forest plots. Trial sequential analysis (TSA) was performed with dichotomous outcomes to minimise the risks of making false positive or negative conclusions, while TSA with continuous outcomes cannot be performed using Stata software.

Sources of heterogeneity were investigated by evaluating whether substantial heterogeneity changed to the absence of substantial heterogeneity based on limitation of studies according to the classifications described in the ‘Data Collection Process and Data Items’ subsection. Subgroup analysis was performed based on limitation of studies according to categories used to investigate sources of heterogeneity and their counterparts. Meta-regression analysis was performed to evaluate whether there were statistically significant differences in the results between the abovementioned categories and their counterparts. Sensitivity analysis was performed based on the exclusion of potential outliers, if any, defined as the studies of which the confidence intervals did not overlap with the confidence intervals of the pooled effects^14^.

### Reporting bias assessment and grading evidence

Publication bias was evaluated using Egger’s test^15^. Language bias was also investigated. The quality of the synthetic evidence was rated based on the Grading of Recommendations, Assessment, Development and Evaluation (GRADE)^16^.

## Results

### Study selection

**Figure 1:**
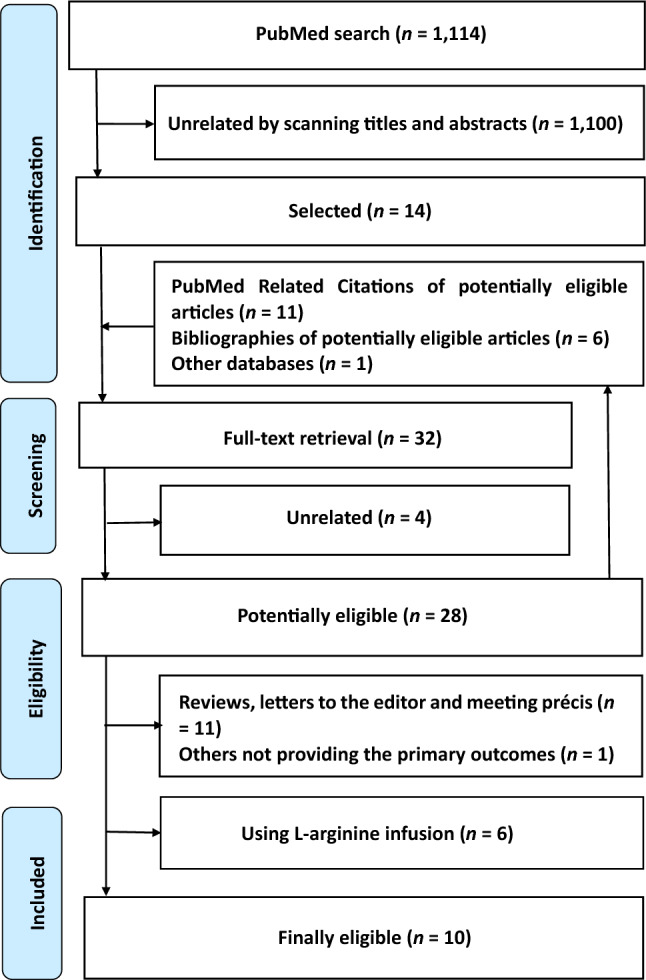
Meta-analysis flow diagram. By excluding 1100 articles determined to be unrelated by scanning the titles and abstracts, 14 articles were selected from 1114 articles identified by PubMed search. With the inclusion of 18 articles by additional investigations, 32 articles were subjected to full-text retrieval. After excluding four unrelated articles, 28 articles were considered to be potentially eligible. Following the exclusion of 18 articles describing studies that did not satisfy the inclusion criteria, 10 articles were finally eligible for the analysis^17–26^. A single article sometimes reported two or more studies among which birth outcomes were different. For example, an article by Camarena Pulido et al. reported a study that evaluated intrauterine growth retardation (IUGR) neonates, another study that evaluated pre-term birth and the other study that evaluated birthweight (Table 1)^17^. Of 10 finally eligible articles, therefore, seven, three, two, two, three, two, two and two studies (i.e., a total of 23 studies) were extracted to evaluate IUGR neonates, pre-term delivery, abortion, infection, respiratory distress syndrome, intracranial hemorrhage, neonatal intensive care unit admission and cesarean section, respectively (Table 1, Figs. 2 and 3 and Supplementary Tables 1 and 2). Eight, two, five and seven studies (i.e., a total of 22 studies) were extracted to evaluate birthweight, birth length, pregnancy duration and Apgar score, respectively. No or only one study that could not be subjected to meta-analysis was extracted to evaluate any other outcome. Therefore, a total of 45 studies involving 5763 mother–neonate pairs in five developing and developed countries in Asia, Europe and Latin America that were extracted from the 10 finally eligible articles were included in this meta-analysis (Table 1, Figs. 2 and 3 and Supplementary Tables 1 and 2).

**Figure 2:**
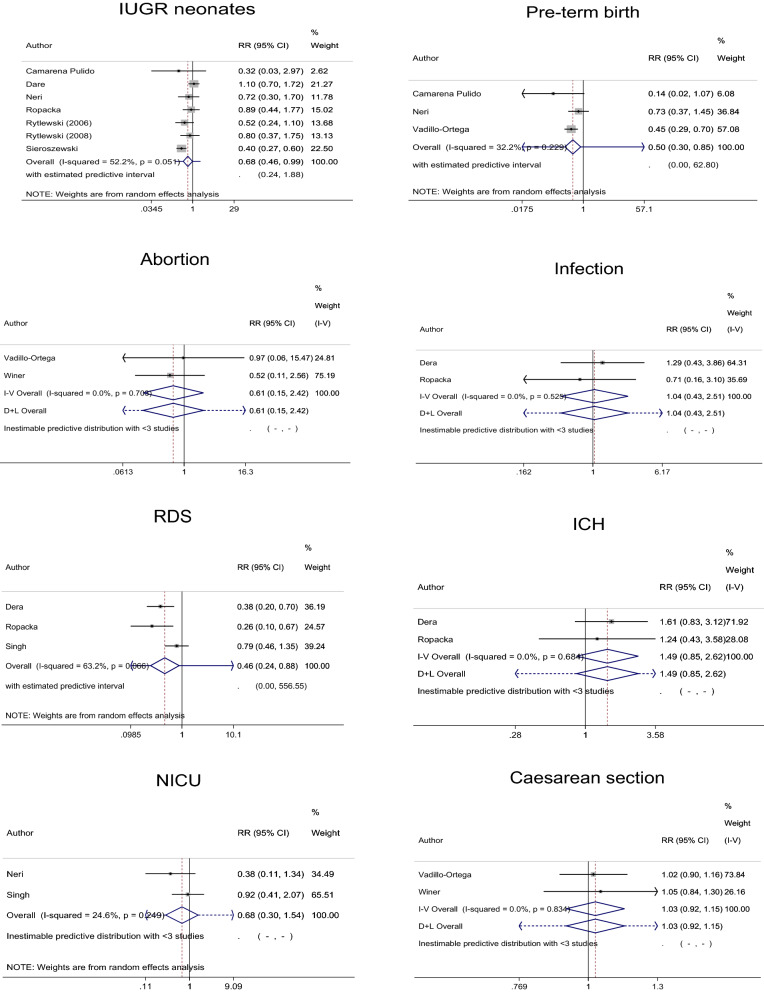
Forest plots of dichotomous birth outcomes. D + L, DerSimonian & Laird; ICH, intracranial haemorrhage; IUGR, intrauterine growth retardation; I-V, inverse variance; NICU, neonatal intensive care unit; RDS, respiratory distress syndrome. The model of DerSimonian & Laird is used when D + L or I-V is not attached to ‘Overall’. In comparison to controls, the oral l-arginine groups showed significantly reduced risks of IUGR neonates, pre-term birth and respiratory distress syndrome (*n* = 7, 3 and 3, respectively) in the total population.

**Figure 3:**
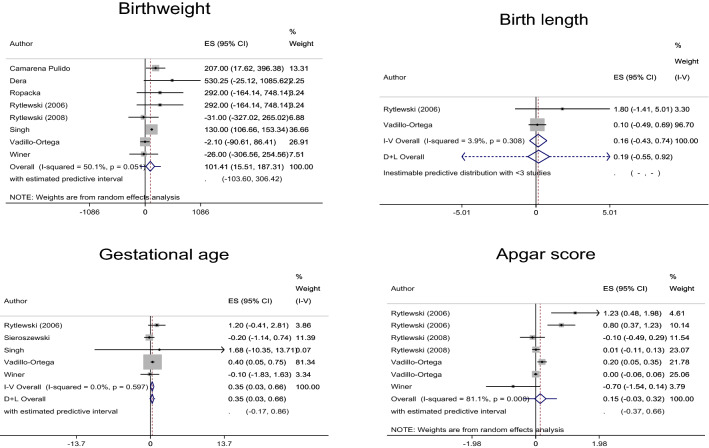
Forest plots of continuous birth outcomes. D + L, DerSimonian & Laird; I-V, inverse variance. The model of DerSimonian & Laird is used when D + L or I-V is not attached to ‘Overall’. In comparison to controls, the oral l-arginine groups showed significantly increased mean birthweight and gestational age (*n* = 8 and 5, respectively) in the total population.

Ten articles were finally eligible for inclusion in the analysis (Fig. 1)^17–26^. A single article sometimes reported two or more studies among which birth outcomes were different. For example, an article by Camarena Pulido et al. reported a study that evaluated IUGR neonates, another study that evaluated pre-term birth and the other study that evaluated birthweight (Table 1)^17^. Of the 10 finally eligible articles, therefore, seven, three, two, two, three, two, two and two studies (i.e., a total of 23 studies) were extracted to evaluate IUGR neonates, pre-term delivery, abortion, infection, RDS, ICH, NICU admission and Caesarean section, respectively (Table 1, Figs. 2 and 3 and Supplementary Tables 1 and 2). Eight, two, five and seven studies (i.e., a total of 22 studies) were extracted to evaluate birthweight, birth length, pregnancy duration and Apgar score, respectively. No or only one study that could not be subjected to meta-analysis was extracted to evaluate any other outcome.

**Table 1:** Characteristics of the included.

Author (Year)	Country	Population	Outcome	Intervention	Control	Start	End
				Content	Content		
Camarena Pulido (2016)	Mexico	High risk of pre-eclampsia	IUGR, PB, BW	l-Arg 3 g/day	Placebo	20 weeks	–
Dare (2007)	Poland	Gestational hypertension and/or IUGR	IUGR, IN, RDS, ICH, BW	l-Arg 3 g/day	Placebo	25–34 weeks	–
Neri (2010)	Italy	Mild chronic hypertension	IUGR, PB, NICUA	l-Arg 4 g/day	Placebo	< 16 weeks	–
Ropacka (2007)	Poland	IUGR	IUGR, IN, RDS, ICH, BW	l-Arg 3 g/day	Placebo	24–36 weeks	Delivery
Rytlewski (2006)	Poland	Pre-eclampsia	IUGR, BW, BL, GA, AS	l-Arg 3 g/day	Placebo	27–31 weeks	Delivery
Rytlewski (2008)	Poland	Threatened pre-term labour	IUGR, BW, AS	l-Arg 3 g/day	Placebo	25–34 weeks	Delivery
Sieroszewski (2004)	Poland	IUGR	IUGR, GA	l-Arg 3 g/day	None	–	–
Singh (2015)	India	Asymmetrical IUGR	RDS, NICUA, BW, GA	l-Arg 3 g/day	None	30–40 weeks	33 weeks –delivery
Vadillo-Ortega (2011)	Mexico	High risk of pre-eclampsia	PB, AB, CA, BW, BL, GA, AS	l-Arg 6.6 g /day + Vitamins	^a^Vitamins	14–32 weeks	Delivery
Winer (2009)	France	^b^Vascular IUGR	IUGR, AB, CA, BW, GA, AS	l-Arg 14 g	Placebo	24–32 weeks	–

*AB* abortion; *Arg* arginine; *AS* Apgar score; *BL* birth length; *BW* birthweight; *CA* Caesarean section; *GA* gestational age; *ICH* intracranial haemorrhage; *IN* infection; *IUGR*, intrauterine growth retardation; *NICUA* neonatal intensive care unit admission; *PB* pre-term birth; *RDS* respiratory distress syndrome.

^a^Vitamins alone were used as controls to accurately evaluate the effects of l-arginine, although another group used a placebo control rather than vitamins.

^b^Foetal abdominal circumference ≤ the 3rd percentile for gestational age and abnormal uterine Doppler sonography.

### Study characteristics

A total of 45 studies, as described in the ‘Study Selection’ subsection, involving 5763 mother–neonate pairs of Asian, Caucasian and Hispanic ethnicities in five developing and developed countries in Asia, Europe and Latin America that were extracted from the 10 finally eligible articles were included in this meta-analysis (Table 1, Figs. 1, 2 and 3 and Supplementary Tables 1 and 2)^17–26^. However, the number of mother–neonate pairs in each of the studies, except those of Vadillo-Ortega et al. (*n* = 450)^25^, was too small or relatively small (range, 43–108) (Table 1). Only two or three studies evaluated pre-term delivery, abortion, infection, RDS, ICH, NICU admission, Caesarean section and birth length (Figs. 2 and 3 and Supplementary Tables 1 and 2), leading to an issue related to ‘imprecision (i.e., uncertainty about the results)’^16^ due to small sample sizes in evaluating all of these outcomes. All of the studies included in the analysis involved women at high risk of pre-eclampsia or with pre-eclampsia or with gestational or mild chronic hypertension, IUGR foetuses or asymmetric or vascular IUGR foetuses or threatened labor (Table 1), i.e., those who are the targets of prenatal oral l-arginine, resulting in no issue related to ‘indirectness of evidence’^16^ in evaluating all of the outcomes.

Twenty-nine studies extracted from seven data sources, three studies extracted from one data source, seven studies extracted from one data source and six studies extracted one data source used 3 g/day l-arginine, 4 g/day l-arginine, 6.6 g/day l-arginine + vitamins and 14 g/day l-arginine as interventions, respectively (Table 1). Thirty-one studies extracted from seven data sources, six studies extracted from two data sources and eight studies extracted from one data source used placebo, none and vitamins as controls, respectively. The timing at which supplementation was started varied among the studies or even among women within the same study in some cases. Twenty-three studies extracted from three data sources and four studies extracted from one data source used supplementation until delivery and until between 33 weeks of gestation and delivery, respectively, but other studies did not clearly describe when the period of supplementation ended.

### Risk of bias in studies

The number of studies not subject to bias related to ‘random sequence generation’^10^, ‘allocation concealment’^10^, and ‘baseline difference’^10^ was much greater than that of studies subject to bias (Supplementary Fig. 1). Although there were some concerns regarding risk of bias in two studies, all of the remaining 43 studies had low risk of bias. Therefore, the overall study quality was good, meaning that there were no issues related to ‘study limitations’^16^ in evaluating overall outcomes (i.e., risk of bias).

### Results of synthesis and certainty assessment

Although there were no significant effects on the risks of abortion, infection, ICH, NICU admission or Caesarean section (*n* = 2 each) in the total population, l-arginine supplementation significantly reduced the risks of IUGR neonates, pre-term birth and RDS (*n* = 7, 3 and 3, respectively) compared to controls in the total population (Fig. 2 and Supplementary Table 1). Although there were no significant effects on the mean birth length or Apgar score (*n* = 2 or 7, respectively) in the total population, l-arginine significantly increased mean birthweight and gestational age (*n* = 8 and 5, respectively) compared to controls in the total population and significantly increased mean Apgar score (*n* = 4) compared to controls in women at high risk of pre-eclampsia or with pre-eclampsia or gestational or mild chronic hypertension (Fig. 3 and Supplementary Table 2). A small grade of risk reduction in IUGR neonates and only small mean increases in gestational age and Apgar score were observed; however, the magnitudes of effects on the risk of pre-term birth and RDS and mean birthweight were sufficiently large, and the 95% confidence intervals of the effects may not have overlapped with values smaller than the thresholds of clinical importance (Supplementary Tables 1 and 2 and Figs. 2 and 3)^16^. Therefore, there were benefits related to ‘the magnitude of an effect’^16^ in evaluating pre-term birth, RDS and birthweight, although not in evaluating IUGR neonates, gestational age and Apgar score. However, 95% prediction intervals revealed no guarantee that the effects of l-arginine on IUGR neonates, pre-term birth, RDS and gestational age would be observed in future studies (Figs. 2 and 3). TSA indicated that the effects of l-arginine on pre-term birth and RDS were true positives but the effect of l-arginine on IUGR neonates was a false positive (Supplementary Fig. 2); the effect on IUGR neonates might have been determined as true negative, but it is more reasonable that it was a false positive because of the green line that should have been located at Z score = 1.96 but was actually located at Z score = 2 and because of the significant reduction in risk of IUGR neonates demonstrated in this meta-analysis. Therefore, the findings of this study were affected by small study effects.

### Investigation of heterogeneity sources

A fixed-effects model and a random-effects model were used to evaluate abortion, infection, ICH, NICU admission, Caesarean section and gestational age due to *I* ^*2*^ = 0% and IUGR neonates, pre-term birth, RDS, birthweight, birth length and Apgar score due to *I*^2^ > 0%, respectively (Figs. 2 and 3 and Supplementary Tables 1 and 2). The sources of the heterogeneities can be explained as follows. All categories that changed substantial heterogeneity to the absence of substantial heterogeity in evaluation of IUGR neonates were due to exclusion of the study by Sieroszewski et al.^23^ (Figs. 2 and 3 and Supplementary Tables 1 and 2). All categories that showed a change from substantial heterogeneity to the absence of substantial heterogeneity in evaluating RDS and all categories, except 3 g/day of l-arginine, that did so in evaluating birthweight were due to exclusion of the study by Singh et al.^24^ The study by Sieroszewski et al.^23^ was the only study that used ‘none’ as a control or had some concerns regarding risk of bias (Table 1 and Supplementary Fig. 1). The study by Singh et al.^24^ was the only study that used ‘none’ as a control (Table 1). A category excluding women at high risk of pre-eclampsia or with pre-eclampsia or gestational or mild chronic hypertension changed substantial heterogeneity to the absence of substantial heterogeity in evaluating Apgar score. However, as mentioned in the ‘Subgroup and Meta-Regression Analysis’ subsection, a category including these subjects changed the effects on Apgar score from non-significant to significant (Supplementary Table 2), which was consistent with evidence indicating that l-arginine reduces blood pressure in pregnancy^27^. Therefore, there were no issues related to ‘inconsistency of results (i.e., unexplained heterogeneity)’^16^ in evaluating all of the outcomes.

### Subgroup and meta-regression analyses

Two categories, i.e., Europe and developed countries that changed the effects on IUGR neonates from significant to non-significant excluded a study by Camarena Pulido et al.^17^ (Table 1, Fig. 2 and Supplementary Table 1), which was the only study that was conducted in a developing country (Mexico). The categories of excluding women at high risk of pre-eclampsia or with pre-eclampsia or gestational or mild chronic hypertension, including women with IUGR foetuses and ‘(probably) no’ response regarding ‘baseline difference’, which changed the effects on IUGR neonates from significant to non-significant excluded a study by Neri et al.^19^ This was the only study that used l-arginine at 4 g/day as an intervention, whereas any of the other studies evaluating IUGR neonates used l-arginine at a dose of 3 g/day as an intervention. The categories of including women at high risk of pre-eclampsia or with pre-eclampsia or gestational or mild chronic hypertension, excluding women with IUGR foetuses, ‘(probably) yes’ response regarding ‘allocation concealment’ and ‘low’ risk-of-bias, which changed the effects on IUGR neonates from significant to non-significant excluded study a study by Sieroszewski et al.^23^, i.e., the most highly weighted study (Fig. 2). The change was possibly due to the small number of studies. These observations indicated possible benefits related to ‘a dose–response gradient’^16^ and ‘an effect of plausible residual confounding’^16^ in evaluating IUGR neonates. A category of placebo control that changed the effects on pre-term birth from significant to non-significant excluded the study by Vadillo-Ortega et al.^25^ (Table 1, Fig. 2 and Supplementary Table 1). This was the only study that used 6.6 g/day of l-arginine + vitamins as an intervention and vitamins as a control, while all of the other studies included to evaluate pre-term birth used l-arginine at a dose of 3 g/day as an intervention and placebo as a control (Table 1). These observations suggested possible benefits related to ‘a dose–response gradient’^16^ and ‘an effect of plausible residual confounding’^16^ in evaluating pre-term birth. A category excluding women at high risk of pre-eclampsia or with pre-eclampsia or gestational or mild chronic hypertension changed the effects on RDS from significant to non-significant (Fig. 2 and Supplementary Table 1), which was consistent with the evidence indicating that l-arginine reduces blood pressure in pregnancy^27^. Therefore, there was no benefit related to ‘a dose–response gradient’^16^, but there was a benefit related to ‘an effect of plausible residual confounding’^16^ in evaluating RDS.

A category of l-arginine at a dose of 4–14 g/day changed the effects on birthweight from significant to non-significant possibly due to the small number of studies. With the exception of developing countries, all categories that showed changes in the effects on birthweight from significant to non-significant excluded the study of Singh et al.^24^ (Table 1, Fig. 3 and Supplementary Table 2). As mentioned in the ‘Investigation of heterogeneity sources’ subsection, this was the only study that used ‘none’ as a control, while all of the other studies included to evaluate birthweight used placebo or vitamins as a control (Table 1). On the other hand, l-arginine at a dose of 3 g/day vs. 4–14 g/day was a confounder and either 3 g/day or 4–14 g/day of l-arginine changed substantial heterogeneity to the absence of substantial heterogeneity in evaluating birthweight (Supplementary Table 2). A category of developing countries showed a change in the effects on birthweight from significant to non-significant, which was possibly due to the small number of studies (Supplementary Table 2). Therefore, there was no benefit relaed to ‘a dose–response gradient’^16^ but a possible benefit related to ‘an effect of plausible residual confounding’^16^ in evaluating birthweight. A category of l-arginine at a dose of 3 g/day that changed the effects on gestational age from significant to non-significant excluded the studies by Vadillo-Ortega et al. and Winer et al.^25,26^ (Table 1, Fig. 3 and Supplementary Table 2), which used 6.6 g/day of l-arginine + vitamins and 14 g/day of l-arginine, respectively (Table 1). Two categories, Europe and developed countries, that changed the effects on gestational age from significant to non-significant excluded the studies by Singh et al. and Vadillo-Ortega et al.^24,25^ conducted in developing countries (India and Mexico, respectively) (Table 1, Fig. 3 and Supplementary Table 2). Two categories excluding women at high risk of pre-eclampsia or with pre-eclampsia or gestational or mild chronic hypertension and including women with IUGR foetuses, between which the same studies were selected, changed the effects on gestational age from significant to non-significant (Fig. 3 and Supplementary Table 2), which was consistent with the evidence indicating that l-arginine reduces blood pressure in pregnancy^27^. These observations indicated benefits related to ‘a dose–response gradient’^16^ and ‘an effect of plausible residual confounding’^16^ in evaluating gestational age. A category of including women at high risk of pre-eclampsia or with pre-eclampsia or gestational or mild chronic hypertension changed the effects on Apgar score from non-significant to significant (Fig. 2 and Supplementary Table 2), which was consistent with the evidence indicating that l-arginine reduces blood pressure in pregnancy^27^. These observations indicated that there was no benefit related to ‘a dose–response gradient’^16^ but a benefit related to ‘an effect of plausible residual confounding’^16^ in evaluating Apgar score. No benefits related to ‘a dose–response gradient’^16^ or ‘an effect of plausible residual confounding’^16^ were identified in evaluating any of the other outcomes, i.e., abortion, infection, ICH, Caesarean section and birth length.

### Reporting biases

Within the availability of *P*-values, Egger’s test did not detect publication bias, as *P*-values (*P* = 0.28–0.95) were much higher than the threshold commonly used for publication bias (*P* = 0.10) (Supplementary Fig. 3)^1^. This indicated that there were no issues related to ‘publication bias’^16^. Despite the limitation of studies to those published in English, none of the countries where the included studies were performed used English as the first language. This also suggested no serious language bias. Sensitivity analysis is described in Supplementary Results.

## Discussion

### Main findings

Oral l-arginine in women with a history of poor pregnancy outcomes was associated with reductions in the risks of IUGR neonates, pre-term birth and RDS and increases in birthweight and gestational age (*n* = 7, 3, 3, 8 and 5, respectively) (Fig. 2 and Supplementary Table 1). Oral l-arginine given to women at high risk of pre-eclampsia or with pre-eclampsia or gestational or mild chronic hypertension was associated with an increase in Apgar score (*n* = 4) (Fig. 3 and Supplementary Tables 1 and 2). On the other hand, oral l-arginine had no effects on abortion, infection, ICH, NICU admission, Caesarean section or birth length (*n* = 2) (Figs. 2 and 3 and Supplementary Table 1 and 2). Based on 95% prediction intervals and TSA, however, the findings of this study were affected by small study effects (Figs. 2 and 3 and Supplementary Fig. 2), while it has been suggested that small study effects are more important in relation to survival data or hazard ratio than risk ratio and mean difference as used in this study^28^. No publication bias was detected (Supplementary Fig. 3).

### Quality of evidence

Despite issues related to ‘indirectness of evidence’^16^ and ‘imprecision’^16^, the evidence to evaluate IUGR neonates, pre-term birth, RDS, birthweight, gestational age and Apgar score was of medium- or high-quality due to benefit(s) related to ‘the magnitude of an effect’^16^, ‘a dose–response gradient’^16^ and/or ‘an effect of plausible residual confounding’^16^. All of these outcomes are those on which prenatal oral l-arginine has favourable effects. As arginine is a semi-essential amino acid, an allowable dose of oral l-arginine is thought to have no serious adverse effects. In addition, oral l-arginine is inexpensive, readily available, can be administered easily and shows its effects relatively rapidly. From both clinical and political perspectives, the outcomes on which parental oral l-arginine has favourable effects are of practical importance. The evidence to evaluate abortion, infection, ICH, NICU admission, Caesarean section and birth length on which prenatal oral l-arginine had no effects was also of medium quality, because there were no clear issues that reduced the quality of evidence, except for those related to ‘imprecision’^16^ (Table 2). Interpretation is described in Supplementary Discussion.

**Table 2:** Quality of evidence.

Outcome	Effects	Issues					Benefits			Quality of evidence
							Magnitude of an effect	A dose-response gradien	An effect of plausible residual confounding	
		Study limitations (risk-of-bias)	Inconsistency of results	Indirectness of evidence	Imprecision	Publication bias				
IUGR neonates	No	−	−	−	+	−	−	+ or unclear	+ or unclear	Medium or high
Pre-term birth	Yes	−	−	−	+	−	+	+ or unclear	+ or unclear	High
Abortion	No	−	−	−	+	^a^NA	^b^NA	^a^NA	^a^NA	Medium
Infection	No	−	−	−	+	^a^NA	^b^NA	^a^NA	^a^NA	Medium
RDS	Yes	−	−	−	+	-	+	-	+	High
ICH	No	−	−	−	+	^a^NA	^b^NA	^a^NA	^a^NA	Medium
NICU admission	No	−	−	−	+	^a^NA	^b^NA	^a^NA	^a^NA	Medium
Caesarean section	No	−	−	−	+	^a^NA	^b^NA	^a^NA	^a^NA	Medium
Birthweight	Yes	−	−	−	+	-	+	-	+ or unclear	High
Birth length	No	−	−	−	+	^a^NA	^b^NA	^a^NA	^a^NA	Medium
Gestational age	Yes	−	−	−	+	−	−	+	+	High
Apgar score	^c^Yes/No	−	−	−	+	−	−	-	+	High

*ICH* intracranial hemorrhage; *IUGR* intrauterine growth retardation; *NA* not applicable; *RDS* respiratory distress syndrome.

^a^”NA” means too small number of studies to allow evaluation of ‘publication bias’^16^, ‘a dose–response gradient’^16^ and ‘an effect of plausible residual confounding’^16^.

^b^”NA” means the irrelevance with ‘magnitude of an effect’^16^ because of no effects on outcomes.

^c^”Yes” in women at high risk of pre-eclampsia or with pre-eclampsia or gestational or mild chronic hypertension but “No” in the total population.

### Results compared to other studies

The results of the present study were consistent with previous reports suggesting a pathophysiological role of l-arginine in placental function, on which pregnancy outcomes may be dependent^3–8^. This was the first meta-analysis to show favourable effects of prenatal oral l-arginine on birth outcomes. As meta-analysis is at the top of the evidence hierarchy, the results of this study were more conclusive than those reported previously. The conclusions that oral l-arginine may improve birth outcomes in pre- and peri-conceptional strategies will also be beneficial for pregnant women, their families, health professionals and policy makers.

### Strengths and limitations of the methodology

The first strength of the present study was that the procedure was based on the guidelines for conducting a meta-analysis^29,30^ and the interpretation of the synthetic evidence was based on Cochrane’s GRADE approach^16^. The second strength was the inclusion of at least medium- or high-quality evidence to evaluate the outcomes on which prenatal oral l-arginine has favourable effects, i.e., pre-term birth, RDS, birthweight, gestational age and Apgar score. The third strength was the use of prediction interval analysis and TSA, which may have revealed small study effects, to provide rigorous conclusions. The first limitation was that a single researcher searched, selected and reviewed the included studies, and studies in languages other than English were excluded. However, efforts were made to minimise missing studies that would be finally eligible by investigating PubMed Related Citations and bibliographic references of potentially eligible articles as well as periodically repeating the process of study selection. A previous meta-analysis performed by this author alone^2^ with the abovementioned efforts to minimise missing studies included more studies than a meta-analysis with the same objective performed by the Cochrane Collaboration^31^ at almost the same time with multiple reviewers who included studies in both English and other languages as well as unpublished studies. Another previous meta-analysis performed by this author alone^32^ with the abovementioned efforts to minimise missing studies also included far more studies than another meta-analysis with the same objective performed by six authors^33^ at almost the same time. Although this meta-analysis included only studies published in English, all of the included studies were conducted in countries where English is not the first language. In addition, the most frequent responses in assessing study quality five times were selected as the final responses to strengthen the reproducibility in study quality assessment. Therefore, this limitation may not have seriously affected study selection. The second limitation was uncertainty regarding whether prenatal l-arginine is more effective than or as effective as any other supplement to improve birth outcomes and whether the results can be extrapolated to ethnicities that were not included in this meta-analysis, e.g., Africans. The third limitation was that further large RCTs are required to overcome issues related to ‘indirectness of the evidence’^16^ and ‘imprecision’^16^ and thus allow stronger recommendations to be made.

## Conclusions

At least medium- or high-quality evidence suggested favourable effects of prenatal oral l-arginine on IUGR neonates, pre-term birth, RDS, birthweight and gestational age in women with a history of poor pregnancy outcomes and on Apgar score in women at high risk of pre-eclampsia or with pre-eclampsia or gestational or mild chronic hypertension. Parental oral l-arginine in these women may be at least moderately recommended to improve birth outcomes, resulting in reduced rates of mortality and morbidity between neonatal and adult periods. However, further studies are required to provide stronger conclusions, partly due to small study effects.

## Supplementary Information

Supplementary Information 1.

Supplementary Tables.

Supplementary Figures.
