# Supplementary material for: Effects of prenatal oral l-arginine on birth outcomes: a meta-analysis
Source: Sci Rep. 2021 Nov 23;11:22748. doi: 10.1038/s41598-021-02182-6 (PMC8610968; doi:10.1038/s41598-021-02182-6)
Supplement: Supplementary file 1 — Supplementary Information 1. [file 41598_2021_2182_MOESM1_ESM.pdf]

Effects of prenatal oral L-arginine on birth outcomes: a meta-analysis

Eita Goto[1,*]

[1]Department of Medicine and Public Health, 1-118 Kamenoi, Meitou-ku, Nagoya 465-0094, Japan

[*]Correspondence to: Dr Eita Goto, Department of Medicine and Public Health, Nagoya Medical Science Research Institute, 1-118 Kamenoi, Meitou-ku, Nagoya 465-0094, Japan TEL: +81-52-702-0941 Email: egoto1@nifty.com ORCID: 0000-0001-5870-8122

Supplementary Information

Supplementary Methods

PubMed search strategy

(maternal OR woman OR women OR mother OR mothers OR pregnancy OR pregnant OR prenatal OR pre-natal OR preconception OR pre-conception OR preconceptional OR pre-conceptional) AND arginine AND (“low birthweight” OR “low birth weight” OR “low birth-weight” OR low-birth-weight OR “small for gestational age” OR “small-for-gestational-age” OR “light for gestational age” OR light-for-gestational-age OR “small for date” OR small-for-date OR “small for dates” OR small-for-dates OR “light for date” OR light-for-date OR “light for dates” OR light-for-dates OR “intrauterine growth restriction” OR “intrauterine-growth-restriction” OR “intra uterine growth restriction” OR “intra-uterine growth restriction” OR “intra-uterine-growth-restriction” OR “intrauterine growth retardation” OR “intrauterine-growth-retardation” OR “intra uterine growth retardation” OR “intra-uterine growth retardation” OR “intra-uterine-growth-retardation” OR “fetal growth restriction” OR “fetal-growth-restriction” OR “fetal growth retardation” OR “fetal-growth-retardation” OR “preterm birth” OR “preterm births” OR “premature birth” OR “premature births” OR stillbirth OR stillbirths OR “still birth” OR “still births” OR still-birth OR still-births OR miscarriage OR miscarriages OR “pregnancy loss” OR pregnancy-loss OR “fatal loss” OR fetal-loss OR abortion OR abortions OR mortality OR death OR underweight OR stunting OR stunt OR wasting OR birthweight OR “ birth weight” OR birth-weight OR ((“crown to rump” OR “crown to coccyx” OR sternal OR foot OR femur OR sole) AND length) OR birth-length OR "birth size" OR birth-size OR ((head OR arm OR chest OR abdominal OR thigh OR calf) AND circumference) OR “biparietal diameter” OR ((subscapular OR bicipital OR biceps R tricipital OR triceps) AND (skinfold OR fold)) OR ((“body mass” OR ponderal OR corpulence) AND index) OR (“weight for age” OR weight-for-age OR “length for age” OR length-for-age OR “weight for height” OR weight-for-height OR “weight for length” OR weight-for-length) OR (“anthropometric measurement” OR “anthropometric measurements” OR anthropometry OR anthropometrics) OR “Apgar score”).

Sensitivity Analysis

A study by Rytlewski (2006) et al. evaluating Apgar score at 1 minute was identified as potential outliers (Figures 2 and 3) [1]. However, the effects on Apgar score did not change from non-significant to significant based on exclusion of this potential outlier—i.e., Apgar score (mean difference = 0.089, 95%CI: –0.069 – 0.244) (Figures 3 and Supplementary Table 2). No outliers were detected in evaluating all the other outcomes.

References

1. Rytlewski, K., Olszanecki, R., Lauterbach, R., Grzyb, A., Basta, A. Effects of oral L-arginine on the foetal condition and neonatal outcome in preeclampsia: a preliminary report, *Basic Clin. Pharmacol. Toxicol.* **99**, 146–152 (2006).

Supplementary Discussion

Interpretations

As mentioned in the 'Study Characteristics' subsection in the Results section, all of the studies included in the analysis involved women at high risk of pre-eclampsia or with pre-eclampsia or with gestational or mild chronic hypertension, IUGR foetuses or asymmetric or vascular IUGR foetuses or threatened labour (Table 1). Therefore, it may not be possible to extrapolate the results to the general population mainly composed of apparently healthy women. As mentioned in the 'Subgroup and Meta-Regression Analyses' subsection of the Results section, the effects on RDS and gestational age changed from significant to non-significant by excluding women at high risk of pre-eclampsia or with pre-eclampsia or gestational or mild chronic hypertension, and the effects on Apgar score changed from non-significant to significant by limiting to them (Table 1 and Supplementary Tables 1 and 2). Therefore, women at high risk of pre-eclampsia or with pre-eclampsia or gestational or mild chronic hypertension would benefit more than others from the use of prenatal oral L-arginine. Furthermore, the effects on IUGR neonates, pre-term birth and gestational age changed from significant to non-significant with the exclusion of a study that used an L-arginine dose of 4 g/day, another study that used an L-arginine dose of 6.6 g/day and those that used doses of 6.6 and 14 g/day of L-arginine, respectively [1–3]. Therefore, an L-arginine dose of 4, 6.6 or 14 g/day would show greater benefit than a dose of 3 g/day. Prenatal oral L-arginine had no serious adverse effects. Vadillo-Ortega et al. reported nausea, dyspepsia, dizziness, palpitation and headache in the group treated with L-arginine + vitamins, but no participants dropped out of their study due to these factors [2]. There were no adverse effects reported in other studies [1, 3 – 10].

There may have been duplicated data to evaluate IUGR neonates, infection, RDS, ICH and birthweight between the studies by Dare et al. and Ropacka et al. [5, 6] (Table 1). However, the duplicated data could not be integrated into one data source, because they could not be accurately divided into duplicated or unduplicated data. Therefore, the results of the present meta-analysis were based on the inclusion of both

studies (Figure 1 and Supplementary Tables 1 and 2). On the other hand, exclusion of the studies by Ropacka et al. or Dera et al. provided the following observations regarding the outcomes that were evaluated including more than two studies [5, 6]. Exclusion of the study by Ropacka et al. or the study by Dera, et al. did not change the effects on IUGR neonates from significant to non-significant (relative risk = 0.644, 95% CI: 0.417, 0.996 or 0.676, 95% CI: 0.464, 0.986, respectively). Exclusion of the study by Ropacka et al. or Dera et al. changed the effects on RDS from significant to non-significant (relative risk = 0.556, 95% CI: 0.270, 1.145 or 0.486, 95% CI: 0.163, 1.446, respectively). Exclusion of the study of Ropacka et al. or Dera et al. also did not change the effects on birthweight from significant to non-significant (mean difference = 95.351, 95% CI: 5.454, 185.249 or 91.455, 95% CI: 8.651, 174.259, respectively). That is, the inclusion of duplicated data did not influence the findings regarding all outcomes, except RDS, on which prenatal oral L-arginine has favourable effects, i.e., IUGR neonates, pre-term birth, birthweight and pregnancy duration in women with a history of poor pregnancy outcomes and Apgar score in women at high risk of pre-eclampsia or with pre-eclampsia or gestational or mild chronic hypertension. Therefore, overall interpretations of the results were not seriously affected by inclusion or exclusion of the duplicated data.

The data extracted from the less weighted studies were required to be added to the data extracted from the highly weighted studies to show that the effects of L-arginine on birthweight were significant. The effects of L-arginine on birthweight changed from significant to non-significant based on the selection of developing countries (Supplementary Table 2) that limited studies to the most, second most and third most weighted, i.e., the studies by Singh et al., Vadillo-Ortega et al. and Camarena Pulido et al. (Figure 3) [2, 4, 10].

References

1. Neri, I. *et al.* L-Arginine supplementation in women with chronic hypertension: impact on blood pressure and maternal and neonatal complications, *J. Matern. Fetal Neonatal Med.* **23**, 1456–1660 (2010).
2. Vadillo-Ortega, F. *et al.* Effect of supplementation during pregnancy with L-arginine and antioxidant vitamins in medical food on pre-eclampsia in high risk population: randomised controlled trial, *BMJ* **342**, d2901 (2011).
3. Winer, N. *et al.* L-Arginine treatment for severe vascular fetal intrauterine growth restriction: a randomized double-bind controlled trial, *Clin. Nutr.* **28**, 243–248 (2009).
4. Camarena Pulido, E.E. *et al.* Efficacy of L-arginine for preventing preeclampsia in high-risk pregnancies: A double-blind, randomized, clinical trial, *Hypertens. Pregnancy*, **35**, 217–225 (2016).
5. Dera, A. *et al.* The effect of L-arginine treatment on the neonatal outcome from pregnancies complicated by intrauterine growth restriction and gestational hypertension, *Archives of Perinatal Medicine*, **13**, 35–39 (2007).

6. Ropacka, M., Kowalska, J., Blumska-Hepner, K. *et al*. The effect of L-arginine on fetal outcome in IUGR fetuses, *Archives of Perinatal Medicine*, **13**, 30–34 (2007).
7. Rytlewski, K., Olszanecki, R., Lauterbach, R. Grzyb, A., Basta, A. Effects of oral L-arginine on the foetal condition and neonatal outcome in preeclampsia: a preliminary report, *Basic Clin. Pharmacol. Toxicol*. **99**, 146–152 (2006).
8. Rytlewski, K. *et al*. Effects of oral L-arginine on the pulsatility indices of umbilical artery and middle cerebral artery in preterm labor, *Eur. J. Obstet. Gynecol. Reprod. Biol.* **138**, 23–28 (2008).
9. Sieroszewski, P., Suzin, J. & Karowicz-Bilińska, A. Ultrasound evaluation of intrauterine growth restriction therapy by a nitric oxide donor (L-arginine), *J. Matern. Fetal Neonatal Med*. **15**, 363–366 (2004).
10. Singh, S. *et al*. Effect of L-arginine on nitric oxide levels in intrauterine growth restriction and its correlation with fetal outcome, *Indian J. Clin. Biochem*. **30**, 298–304 (2015).
