# Supplementary material for: Effects of prenatal oral l-arginine on birth outcomes: a meta-analysis
Source: Sci Rep. 2021 Nov 23;11:22748. doi: 10.1038/s41598-021-02182-6 (PMC8610968; doi:10.1038/s41598-021-02182-6)
Supplement: Supplementary file 2 — Supplementary Tables. [file 41598_2021_2182_MOESM2_ESM.pdf]

Effects of prenatal oral L-arginine on birth outcomes: a meta-analysis

Eita Goto[1,*]

[1]Department of Medicine and Public Health, 1-118 Kamenoi, Meitou-ku, Nagoya 465-0094, Japan

[*]Correspondence to: Dr Eita Goto, Department of Medicine and Public Health, Nagoya Medical Science Research Institute, 1-118 Kamenoi, Meitou-ku, Nagoya 465-0094, Japan TEL: +81-52-702-0941 Email: egoto1@nifty.com ORCID: 0000-0001-5870-8122

Supplementary Tables

Supplementary Table 1. Results of meta-analysis and subgroup and meta-regression analysis (dichotomous outcomes)

**IUGR neonates (499 mother–neonate pairs)**	Relative risk			Meta-regression
Category (number of studies)	Mean	95% CI	I^2 (%)	*P*- value
Total population (*n* = 7)	0.676	0.464, 0.986	52.2	-
Region				
Europe (*n* = 6)	0.691	0.466, 1.024	58.8	0.55
Developed countries (*n* = 6)	0.691	0.466, 1.024	58.8	0.55
Population				
(High risk of) pre-eclampsia/hypertension: include (*n* = 4)	0.850	0.600, 1.202	22.0	0.51
(High risk of) pre-eclampsia/hypertension: exclude (*n* = 3)	0.613	0.350, 1.071	59.6	-
IUGR foetuses: include (*n* = 3)	0.72	0.361, 1.436	82.5	0.95
IUGR foetuses: exclude (*n* = 4)	0.641	0.414, 1.005	0.0	-
Intervention				
3 g/day (*n* = 6)	0.656	0.513, 0.839	60.0	0.89
Control				
Placebo (*n* = 6)	0.849	0.636, 1.133	0.0	0.14
Study quality				
Random sequence generation: (probably) yes (*n* = 7)	0.676	0.464, 0.986	52.2	-
Allocation concealment: (probably) yes (*n* = 6)	0.849	0.636, 1.133	0.0	0.14
Baseline difference: (probably) no (*n* = 6)	0.670	0.435, 1.034	60.0	0.89
Risk of bias: low (*n* = 6)	0.849	0.636, 1.133	0.0	0.14

**Pre-term birth (625 mother–neonate pairs)**	Relative risk			Meta-regression
Category (number of studies)	Mean	95% CI	I^2 (%)	*P*- value
Total population (*n* = 3)	0.499	0.345, 0.845	32.2	-
Region				
Latin America (*n* = 2)	0.426	0.275, 0.659	18.3	0.68

Developing countries (*n* = 2)	0.426	0.275, 0.659	18.3	0.68
Population				
(High risk of) pre-eclampsia/hypertension: include (*n* = 3)	0.499	0.345, 0.845	32.2	-
IUGR foetuses: exclude (*n* = 3)	0.499	0.345, 0.845	32.2	-
Intervention				
4 – 14 g/day (*n* = 2)	0.521	0.358, 0.756	27.6	0.79
Control				
Placebo (*n* = 2)	0.413	0.081, 2.119	60.5	-
Study quality				
Random sequence generation: (probably) yes (*n* = 3)	0.499	0.345, 0.845	32.2	-
Allocation concealment: (probably) yes (*n* = 3)	0.499	0.345, 0.845	32.2	-
Baseline difference: (probably) no (*n* = 2)	0.426	0.275, 0.845	18.3	-
Risk of bias: low (*n* = 3)	0.499	0.345, 0.845	32.2	-

**Abortion (493 mother–neonate pairs)**	Relative risk			Meta-regression
Category (number of studies)	Mean	95% CI	I^2 (%)	*P*- value
Total population (*n* = 2)	0.616	0.157, 2.415	0.0	-
Intervention				
Amount: 4 – 14 g/day (*n* = 2)	0.616	0.157, 2.415	0.0	-
Study quality				
Random sequence generation: (probably) yes (*n* = 2)	0.616	0.157, 2.415	0.0	-
Allocation concealment: (probably) yes (*n* = 2)	0.616	0.157, 2.415	0.0	-
Baseline difference: (probably) no (*n* = 2)	0.616	0.157, 2.415	0.0	-
Risk of bias: low (*n* = 2)	0.616	0.157, 2.415	0.0	-

**Infection (110 mother–neonate pairs)**	Relative risk			Meta-regression
Category (number of studies)	Mean	95% CI	I^2 (%)	*P*- value
Total population (*n* = 2)	1.039	0.431, 2.508	0.0	-
Region				
Europe (*n* = 2)	1.039	0.431, 2.508	0.0	-
Developed countries (*n* = 2)	1.039	0.431, 2.508	0.0	-
Population				
IUGR foetuses: include (*n* = 2)	1.039	0.431, 2.508	0.0	-
Intervention				
Energy before birth: 3 kg (*n* = 2)	1.039	0.431, 2.508	0.0	-
Control				
Placebo (*n* = 2)	1.039	0.431, 2.508	0.0	-
Study quality				

Random sequence generation: (probably) yes (n = 2)	1.039	0.431, 2.508	0.0	-
Allocation concealment: (probably) yes (n = 2)	1.039	0.431, 2.508	0.0	-
Baseline difference: (probably) no (n = 2)	1.039	0.431, 2.508	0.0	-
Risk of bias: low (n = 2)	1.039	0.431, 2.508	0.0	-

**RDS (160 mother–neonate pairs)**	Relative risk			Meta-regression
Category (number of studies)	Mean	95% CI	I^2 (%)	P- value
Total population (n = 3)	0.460	0.241, 0.878	63.2	-
Region				
Europe (n = 2)	0.338	0.202, 0.567	0.0	0.26
Developed countries (n = 2)	0.338	0.202, 0.567	0.0	0.26
Population				
(High risk of) pre-eclampsia/hypertension: exclude (n = 2)	0.486	0.163, 1.446	75.0	0.83
IUGR foetuses: include (n = 3)	0.460	0.241, 0.878	63.2	-
Intervention				
Amount: 3 g/day (n = 3)	0.460	0.241, 0.878	63.2	-
Control				
Placebo (n = 2)	0.338	0.202, 0.567	0.0	0.27
Study quality				
Random sequence generation: (probably) yes (n = 3)	0.460	0.241, 0.878	63.2	-
Allocation concealment: (probably) yes (n = 3)	0.460	0.241, 0.878	63.2	-
Baseline difference: (probably) no (n = 3)	0.460	0.241, 0.878	63.2	-
Risk of bias: low (n = 3)	0.460	0.241, 0.878	63.2	-

**ICH (110 mother–neonate pairs)**	Relative risk			Meta-regression
Category (number of studies)	Mean	95% CI	I^2 (%)	P- value
Total population (n = 2)	1.494	0.852, 2.620	0.0	-
Region				
Europe (n = 2)	1.494	0.852, 2.620	0.0	-
Developed countries (n = 2)	1.494	0.852, 2.620	0.0	-
Population				
IUGR foetuses: include (n = 2)	1.494	0.852, 2.620	0.0	-
Intervention				
Amount: 3 g/day (n = 3)	1.494	0.852, 2.620	0.0	-
Control				
Placebo (n = 2)	1.494	0.852, 2.620	0.0	-
Study quality				
Random sequence generation: (probably) yes (n = 2)	1.494	0.852, 2.620	0.0	-

Allocation concealment: (probably) yes (n = 2)	1.494	0.852, 2.620	0.0	-
Baseline difference: (probably) no (n = 2)	1.494	0.852, 2.620	0.0	-
Risk of bias: low (n = 2)	1.494	0.852, 2.620	0.0	-

**NICU admission (129 mother–neonate pairs)**	Relative risk			Meta-regression
Category (number of studies)	Mean	95% CI	I^2 (%)	P- value
Total population (n = 2)	0.638	0.302, 1.534	24.6	-
Study quality				
Random sequence generation: (probably) yes (n = 2)	0.638	0.302, 1.534	24.6	-
Allocation concealment: (probably) yes (n = 2)	0.638	0.302, 1.534	24.6	-
Baseline difference: (probably) no (n = 2)	0.638	0.302, 1.534	24.6	-
Risk of bias: low (n = 2)	0.638	0.302, 1.534	24.6	-

**Caesarean section (493 mother–neonate pairs)**	Relative risk			Meta-regression
Category (number of studies)	Mean	95% CI	I^2 (%)	P- value
Total population (n = 2)	1.027	0.919, 1.147	0.0	-
Study quality				
Random sequence generation: (probably) yes (n = 2)	1.027	0.919, 1.147	0.0	-
Allocation concealment: (probably) yes (n = 2)	1.027	0.919, 1.147	0.0	-
Baseline difference: (probably) no (n = 2)	1.027	0.919, 1.147	0.0	-
Risk of bias: low (n = 2)	1.027	0.919, 1.147	0.0	-

CI, confidence interval; ICH, intracranial hemorrhage; IUGR, intrauterine growth retardation; NICU, neonatalintensive care unit; RDS, respiratory distress syndrome.

Supplementary Table 2. Results of meta-analysis and subgroup and meta-regression analysis (continuous outcomes)

**Birthweight (g) (755 mother–neonate pairs)**	Difference			Meta-regression
Category (number of studies)	Mean	95% CI	I^2 (%)	P- value
Total population (n = 8)	101.411	15.51, 187.32	50.1	-
Region				
Europe (n = 5)	103.931	–60.54, 268.39	23.0	0.86
Latin America (n = 2)	85.002	–11.04, 287.04	74.0	0.59
Developing countries (n = 3)	97.667	–7.24, 202.58	77.2	0.87
Developed countries (n = 5)	103.931	–60.54, 268.39	23.0	-
Population				
(High risk of) pre-eclampsia/hypertension: include (n = 4)	160.587	–38.09, 359.27	62.1	0.70

(High risk of) pre-eclampsia/hypertension: exclude (*n* = 4)	128.370	105.22, 121.53	0.0	-
IUGR foetuses: include (*n* = 4)	130.051	106.85, 153.26	18.2	0.51
IUGR foetuses: exclude (*n* = 4)	38.151	–38.15, 114.46	42.8	-
Intervention				
3 g/day (*n* = 6)	131.676	108.66, 154.69	0.0	0.023
4 – 14 g/day (*n* = 2)	–4.263	–88.67, 80.14	0.0	-
Control				
Placebo (*n* = 6)	148.239	24.07, 272.41	14.4	0.85
Study quality				
Random sequence generation: (probably) yes (*n* = 8)	101.411	15.51, 187.32	50.1	-
Allocation concealment: (probably) yes (*n* = 8)	101.411	15.51, 187.32	50.1	-
Baseline difference: (probably) no (*n* = 8)	101.411	15.51, 187.32	50.1	-
Risk of bias: low (*n* = 8)	101.411	15.51, 187.32	50.1	-

**Birth length (cm) (511 mother–neonate pairs)**	Difference			Meta-regression
Category (number of studies)	Mean	95% CI	I^2 (%)	*P*- value
Total population (*n* = 2)	0.156	–0.427, 0.739	3.9	-
Population				
(High risk of) pre-eclampsia/hypertension: include (*n* = 2)	0.156	–0.427, 0.739	3.9	-
IUGR foetuses: exclude (*n* = 2)	0.156	–0.427, 0.739	3.9	-
Study quality				
Random sequence generation: (probably) yes (*n* = 2)	0.156	–0.427, 0.739	-	-
Allocation concealment: (probably) yes (*n* = 2)	0.156	–0.427, 0.739	-	-
Baseline difference: (probably) no (*n* = 2)	0.156	–0.427, 0.739	-	-
Risk of bias: low (*n* = 2)	0.156	–0.427, 0.739	-	-

**Gestational age (weeks) (723 mother–neonate pairs)**	Difference			Meta-regression
Category (number of studies)	Mean	95% CI	I^2 (%)	*P*- value
Total population (*n* = 5)	0.347	0.030, 0.663	0.0	-
Region				
Europe (*n* = 3)	0.109	–0.625, 0.843	10.5	0.64
Developing countries (*n* = 2)	0.402	0.050, 0.752	0.0	0.64
Developed countries (*n* = 3)	0.109	–0.625, 0.843	10.5	-
Population				
(High risk of) pre-eclampsia/hypertension: include (*n* = 2)	0.436	0.093, 0.779	0.0	0.28
(High risk of) pre-eclampsia/hypertension: exclude (*n* = 3)	–0.169	–0.992, 0.655	0.0	-
IUGR foetuses: include (*n* = 3)	–0.169	–0.992, 0.655	0.0	0.28
IUGR foetuses: exclude (*n* = 2)	0.436	0.093, 0.779	0.0	-

Intervention				
3 g/day (*n* = 3)	0.162	–0.647, 0.970	10.2	0.85
4 – 14 g/day (*n* = 2)	0.380	0.036, 0.724	0.0	-
Study quality				
Random sequence generation: (probably) yes (*n* = 5)	0.347	0.030, 0.663	0.0	-
Allocation concealment: (probably) yes (*n* = 4)	0.417	0.081, 0.753	0.0	0.31
Baseline difference: (probably) no (*n* = 5)	0.346	0.030, 0.663	0.0	-
Risk of bias: low (*n* = 4)	0.417	0.081, 0.753	0.0	0.31

**Apgar score (1155 mother–neonate pairs)**	Difference			Meta-regression
Category (number of studies)	Mean	95% CI	I^2 (%)	*P*- value
Total population (*n* = 7)	0.146	–0.031, 0.323	81.1	-
Region				
Europe (*n* = 5)	0.247	–0.212, 0.705	84.1	0.77
Latin America (*n* = 2)	0.088	–0.107, 0.282	83.8	0.77
Developing countries (*n* = 2)	0.088	–0.107, 0.282	83.8	0.77
Developed countries (*n* = 5)	0.247	–0.212, 0.705	84.1	-
Population				
(High risk of) pre-eclampsia/hypertension: include (*n* = 4)	0.375	0.065, 0.684	89.3	0.14
(High risk of) pre-eclampsia/hypertension: exclude (*n* = 3)	–0.012	–0.123, 0.100	31.4	-
IUGR foetuses: exclude (*n* = 6)	–0.034	–0.012, 0.081	82.7	0.21
Intervention				
3 g/day (*n* = 4)	0.401	–0.090, 0.892	86.4	0.30
4 – 14 g/day (*n* = 3)	0.045	–0.168, 0.258	77.8	-
Time of Apgar score				
1 minute after delivery (*n* = 3)	0.319	–0.164, 0.802	79.2	0.57
5 minutes after delivery (*n* = 4)	0.086	–0.120, 0.292	80.9	-
Study quality				
Random sequence generation: (probably) yes (*n* = 7)	0.146	–0.031, 0.323	81.1	-
Allocation concealment: (probably) yes (*n* = 7)	0.146	–0.031, 0.323	81.1	-
Baseline difference: (probably) no (*n* = 7)	0.146	–0.031, 0.323	81.1	-
Risk of bias: low (*n* = 7)	0.146	–0.031, 0.323	81.1	-

CI, confidence interval; IUGR, intrauterine growth retardation.
