# Supplementary material for: Effects of prenatal oral l-arginine on birth outcomes: a meta-analysis
Source: Sci Rep. 2021 Nov 23;11:22748. doi: 10.1038/s41598-021-02182-6 (PMC8610968; doi:10.1038/s41598-021-02182-6)
Supplement: Supplementary file 3 — Supplementary Figures. [file 41598_2021_2182_MOESM3_ESM.pdf]

Effects of prenatal oral L-arginine on birth outcomes: a meta-analysis

Eita Goto[1,*]

[1]Department of Medicine and Public Health, 1-118 Kamenoi, Meitou-ku, Nagoya 465-0094, Japan

[*]Correspondence to: Dr Eita Goto, Department of Medicine and Public Health, Nagoya Medical Science Research Institute, 1-118 Kamenoi, Meitou-ku, Nagoya 465-0094, Japan TEL: +81-52-702-0941 Email: egoto1@nifty.com ORCID: 0000-0001-5870-8122

Supplementary Figures

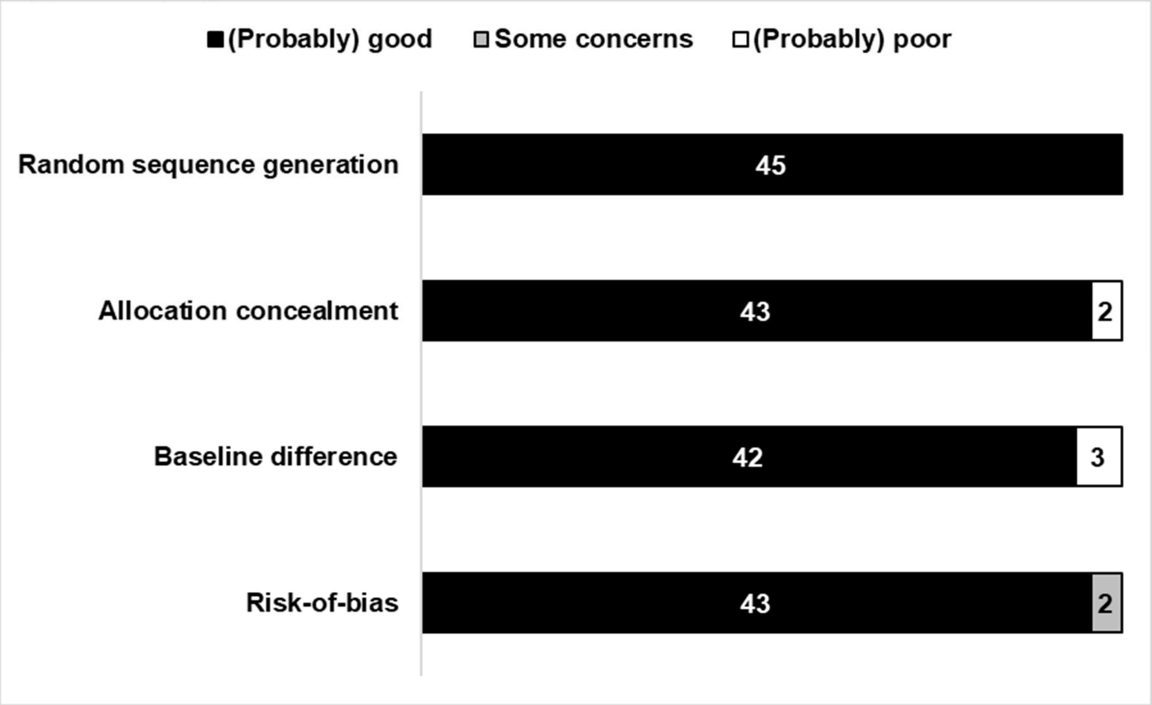

Supplementary Figure 1. Results of study quality assessment
Black bars and white bars represent the numbers of studies in which biases related to random sequence generation, allocation concealment and baseline difference were well controlled and poorly controlled, respectively, and risk of bias was low and high, respectively. Gray bars represent the numbers of studies that had some concerns regarding risk of bias.

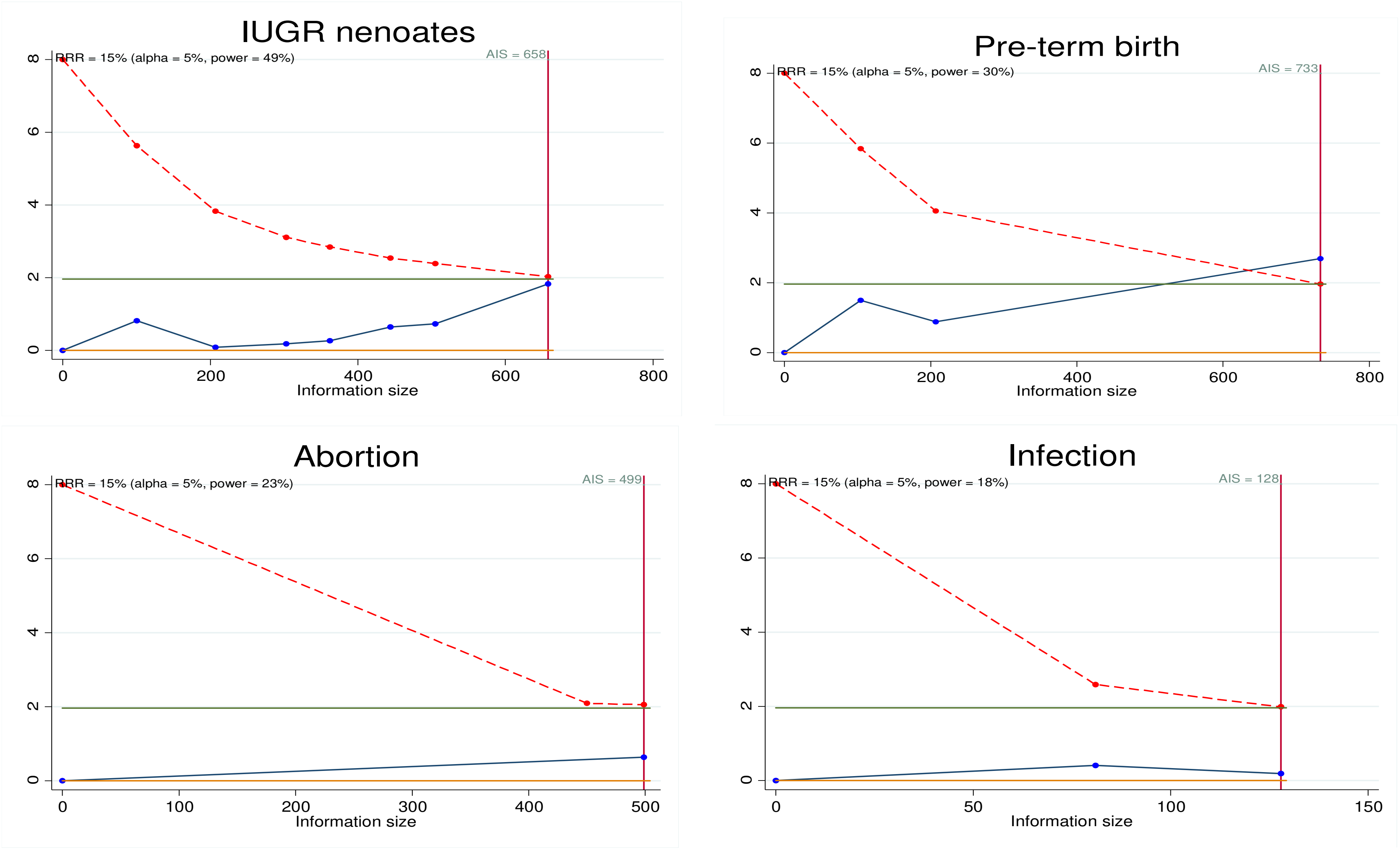
IUGR nenoates
RRR = 15% (alpha = 5%, power = 49%)
AIS = 658
Information size
Pre-term birth
RRR = 15% (alpha = 5%, power = 30%)
AIS = 733
Information size
Abortion
RRR = 15% (alpha = 5%, power = 23%)
AIS = 499
Information size
Infection
RRR = 15% (alpha = 5%, power = 18%)
AIS = 128
Information size

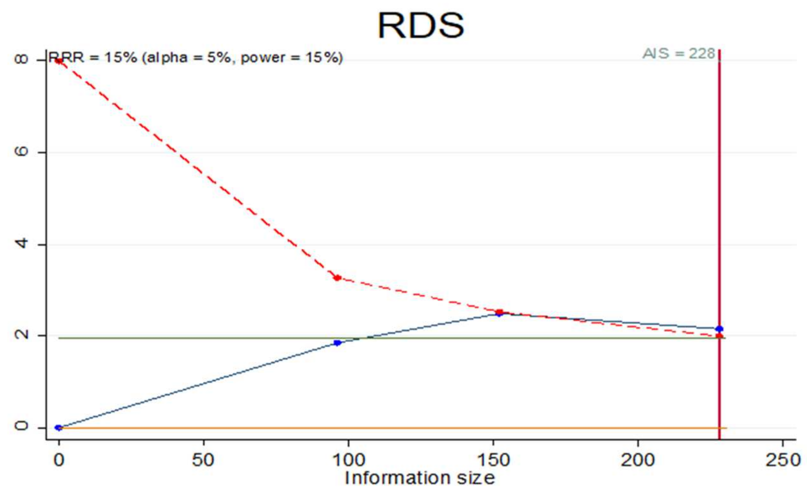
RDS
RRR = 15% (alpha = 5%, power = 15%)
AIS = 228
0
2
4
6
8
0
50
100
150
200
250
Information size

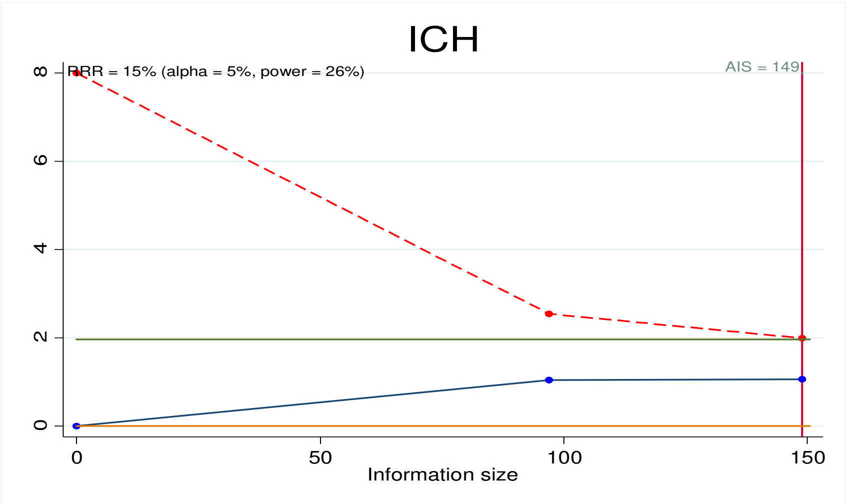
ICH
RRR = 15% (alpha = 5%, power = 26%)
AIS = 149
0
2
4
6
8
0
50
100
150
Information size

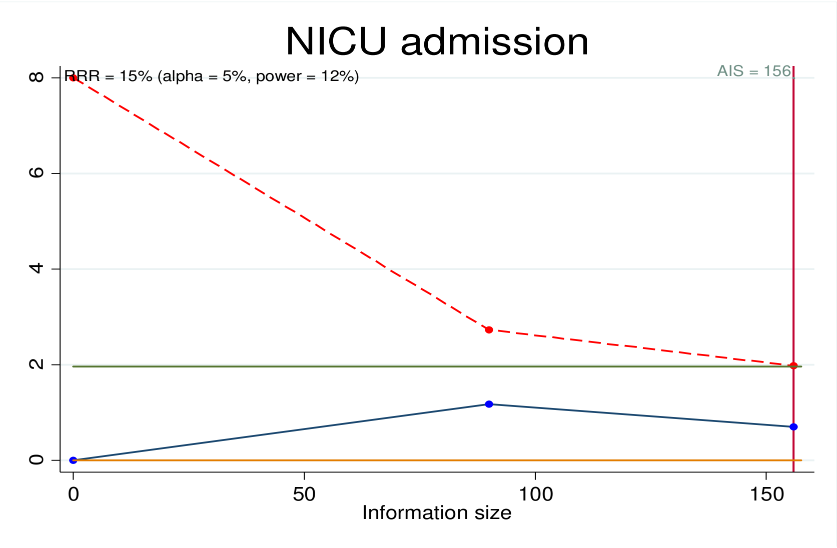
NICU admission
RRR = 15% (alpha = 5%, power = 12%)
AIS = 156
0
2
4
6
8
0
50
100
150
Information size

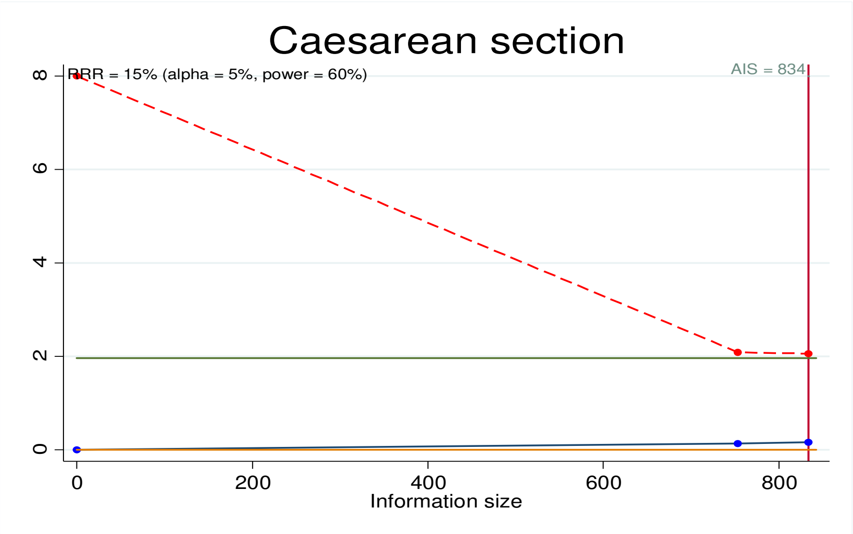
Caesarean section
RRR = 15% (alpha = 5%, power = 60%)
AIS = 834
0
2
4
6
8
0
200
400
600
800
Information size

Supplementary Figure 2. Results of trial sequential analysis with dichotomous outcomes
AIS, accrued information size; ICH, intracranial haemorrhage; IUGR, intrauterine growth retardation; NICU, neonatal intensive care unit; RDS, respiratory distress syndrome; RRR, relative risk reduction.
The effects of L-arginine on preterm birth and RDS were true positives but the effect of L-arginine on IUGR neonates was a false positive; the effect on IUGR neonates might have been determined as true negative, but it is more reasonable that it was a false positive because of the green line that should have been located at Z score = 1.96 but was actually located at Z score = 2 and because of the significant reduction in the risk of IUGR neonates demonstrated in this meta-analysis.

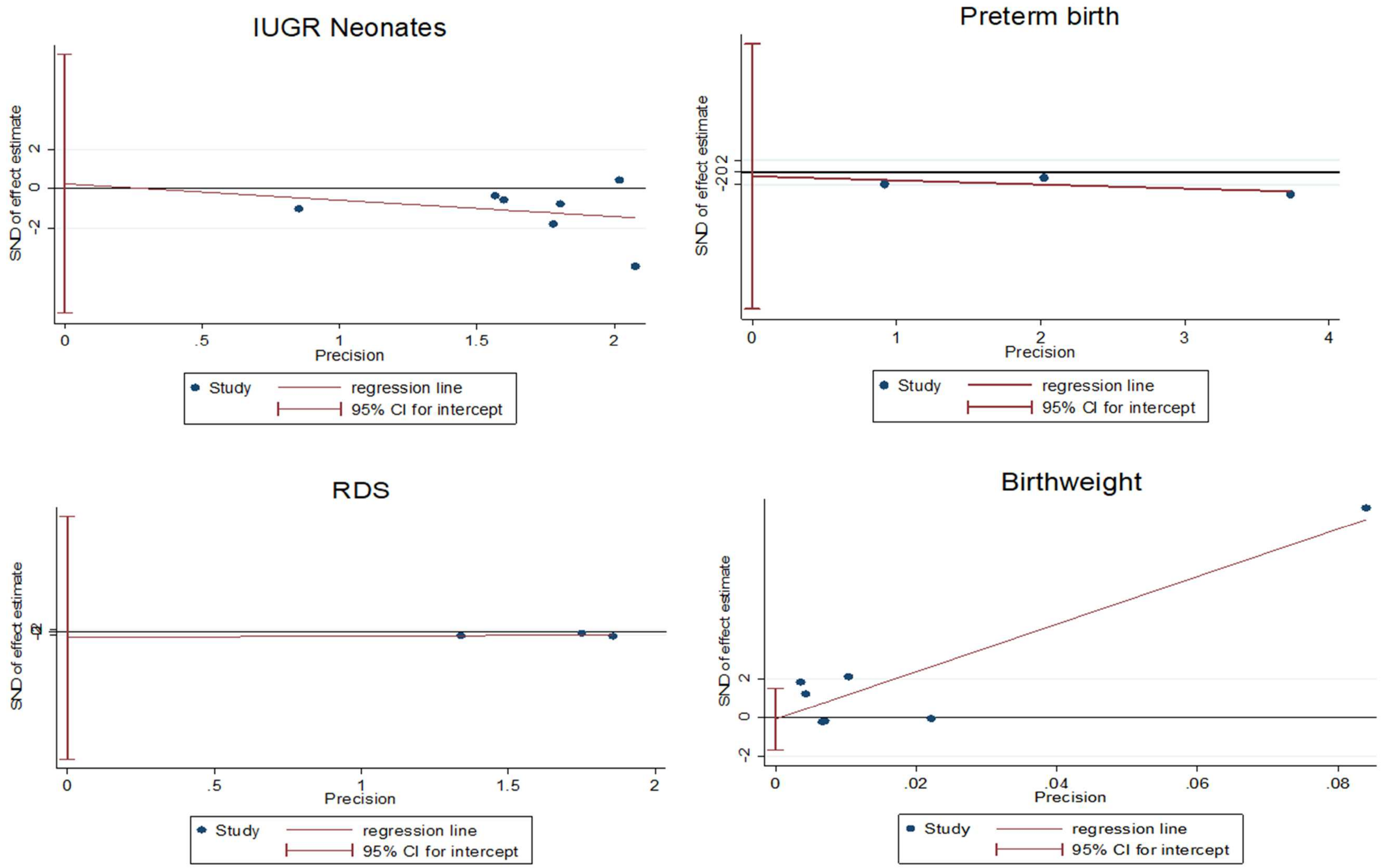
IUGR Neonates
SND of effect estimate
-2
0
2
0
.5
1
1.5
2
Precision
Study
regression line
95% CI for intercept
Preterm birth
SND of effect estimate
0
1
2
3
4
Precision
Study
regression line
95% CI for intercept
RDS
SND of effect estimate
0
.5
1
1.5
2
Precision
Study
regression line
95% CI for intercept
Birthweight
SND of effect estimate
-2
0
2
0
.02
.04
.06
.08
Precision
Study
regression line
95% CI for intercept

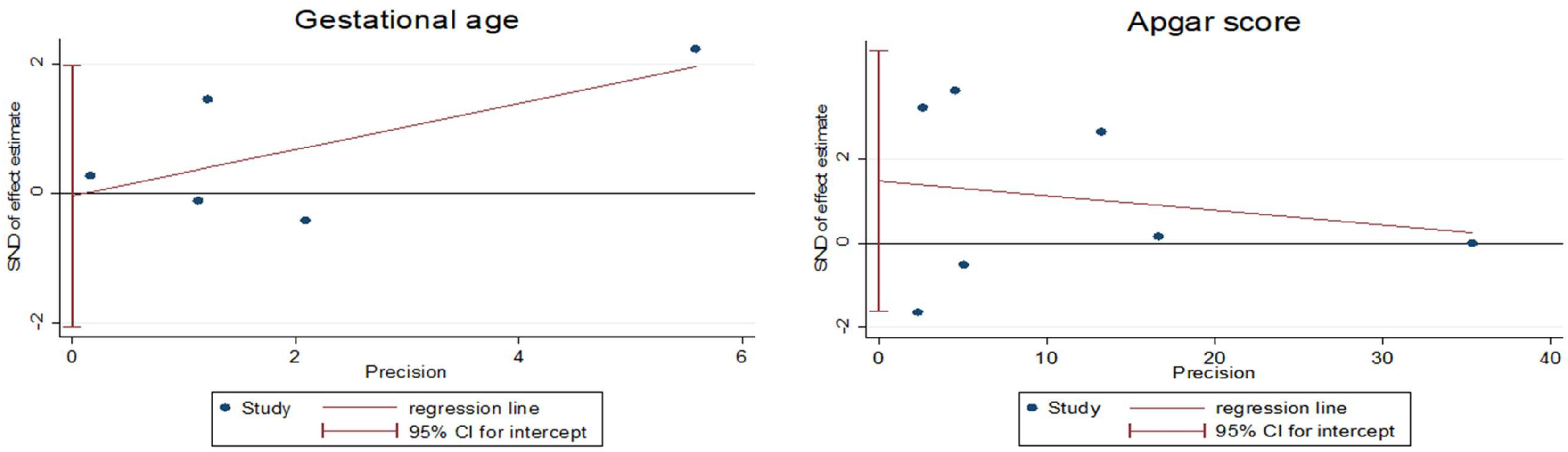

Supplementary Figure 3. Results of publication bias assessment (Egger's regression asymmetry plot test). CI, confidence interval; IUGR, intrauterine growth retardation; RDS, respiratory distress syndrome; SND, standard normal deviate. 'Let (t_i, v_i), $i = 1,\ldots,k$, be the estimated effect sizes and sample variances from k studies. Define the standardized effect size as $t^*_i = t_i/v_i^{1/2}$, the precision as $s^{-1} = 1/v_i^{1/2}$ and the weight as $w_i = 1/v_i$. Egger designates t^* as a *standard normal deviate* [1]. Fit t^* to s^{-1} using standard weighted linear regression with weights w and linear equation: $t^* = a + bs^{-1}$. A significant deviation from zero of the estimated intercept, $\hat{a}$, is interpreted as providing evidence of asymmetry in the funnel plot and evidence of publication bias' [2].

There was no publication bias in the data used to evaluate intrauterine growth retardation neonates, preterm birth, respiratory distress syndrome, birthweight, gestational age and Apgar score between L-arginine group and control group, because zero was within the 95% confidence interval for intercept of the regression line. The upper and lower limits of the 95% confidence intervals were not close to zero, indicating no weak evidence for the presence of publication bias (unlikeliness of the results' being false positive). *P*-values were unavailable for other outcomes due to small numbers of studies (= 2).

References

1. Egger, M., Davey Smith, G., Schneider, M., Minder, C. Bias in meta-analysis detected by a simple, graphical test. *BMJ* **315**, 629–634 (1997).
2. Steichen, T.J. Tests for publication bias in meta-analysis. *Stata Technical Bull*. **41**, 9–15 (1998).
